# Report of the Committee on Practical Medicine

**Published:** 1856-08

**Authors:** Samuel Thompson

**Affiliations:** Albion, Edwards County, Ill.


THE NORTH-WESTERN

MEDICAL AND SURGICAL JOURNAL.

(NEW SERIES.)

VOL. V. AUGUST, 1856. NO. 8.

### ORIGINAL COMMUNICATIONS.

### *Report of the Committee on Practical Medicine.* By SAMUEL THOMPSON, M.D., of Albion, Edwards County, Ill. Presented to the State Medical Society.

The Committee on Practical Medicine and Epidemics, of the Illinois State Medical Society, appointed in June, 1855, submit the following imperfect report:—

Under any circumstances the duties of this committee are arduous and perplexing; but, in the present instance, that difficulty has been greatly enhanced by the unfortunate delay in publishing the Transactions of the Society for last year: so that your committee were ignorant of the duties expected of them till the latter end of March. A question even arose to part of them, whether *any* report could be attempted; but it was considered, that, though no "specific pledge," had been given, a report of some kind should be forthcoming—that an understood pledge was always in existence, that members of this Society, to whom duties are delegated, shall, one or other of them, to the best of their abilities, perform the duties required. Therefore, upon receiving notice of appointment, circulars were printed and forwarded to various members of the profession in different parts of the State, soliciting facts and information. But, as in 1851, we did not receive fifty replies to hundreds of circulars; and, as in 1855, the committee (Dr. Roe, chairman) state that they received *a few, a very few replies:* so it has been this present year, we have received but

*six* letters, and are, therefore, in our report, confined to the materials furnished by these six gentlemen—to what the medical journals supply—and to the personal experience of your committee. And here we must remark, that, while grateful to those gentlemen for *any* assistance, yet there is a sad deficiency in some of their reports of those details, of which *alone* sound statistics can be formed, and upon which statistics *only*, so formed, sound deductions can be based: and we presume it is not claiming too high an object for the reports of the various committees appointed by this Society to assume, that they are intended to be, and ought to be, *our State's contributions* to the wealth of our profession.

Now, when we can scarcely open a volume of periodical or systematic medical literature without finding such diversities of opinion in reference to the *one* subject of typhoid fever alone, as actually to render its absolute identification a matter of no small difficulty; when, in reference to its diagnosis, the most eminent and experienced in our profession disagree, or, to use the words of Prof. Davis, "there is no agreement among writers as to what shall be called typhoid fever," (see *N. W. Journal*, Nov., 1853, p. 451;) and when the first question a man asks himself, when reading an article under that caption, is, Well, but what does the writer understand by the term? when such is the condition of the professional mind, it becomes doubly important that our records should not be of typhoid fever, (we name typhoid fever only as an example,) for it is a name that defines nothing, but of certain symptoms, conditions, and effects, constituting the disease we so define. Thus, by collecting from many quarters the many descriptions of a disease occurring in many places, we should find out whether we were all really talking about the same thing, or of *various* disorders under one common name. Hence, was the question in our circular worded—"Has typhoid fever occurred in your neighborhood?—Describe what were the symptoms and treatment." We considered that many descriptions, from many distant places, would, by comparison, have shewn the identity or diversity of the things spoken of. But one gentleman, who has supplied quite an important contribution to this report, says "typhoid fever occurred here quite malignantly in the year

1848, &c.," and goes on to give the *treatment*. But, your committee had hoped to have collected facts—not opinions; details—not conclusions. The celebrated John Armstrong asserted, that there were more false facts than false theories in medicine; and we believe it: and there is no doubt but that loose observations, careless records, and careless generalizations can lead to nothing but error. But it is careful observations recorded, not remembered, that are needed as contributions to medical science. It is very true, that it is very difficult for a physician, in daily, laborious, and extensive practice to examine carefully, amid all the noises and confusion of a country log-cabin, with the cooking, and creaking, and squealing around, and as difficult to record carefully such observations when made; but they are necessary, and will well repay the labor. And, now, having let off our ill-humor in scolding, and admonition, and advice, *all* unpalatable doses, we will endeavor to arrange into something like form the scanty materials we have before us.

We begin with that class of diseases which, upon the democratic principle of the rights of majorities, has a right to precedence, namely, Periodic Fevers, which appear to have been more extensively prevalent during 1855 than at any previous period. Dr. Harris, of Ottawa, states, that by far the most usual diseases in his vicinity are those of a malarious origin, including remittents, intermittents, and pernicious fevers, and occurring chiefly during the months of August, September, October, and November; but that, during the last two years, they have occurred at all seasons of the year, especially during the past winter. He places his chief dependence in the treatment on quinine, though occasionally cases occurred which would not yield to quinine, and in these cases he has been in the habit of combining several anti-periodics, as follows:—Quinine, salacine, bebeerine, piperine, sulph. zinci, aa, gr. x., in pills x., one every two hours; to be repeated in some cases the next day. He prefers giving quinine in solution, thus:—Quinine, ℈j.; morph. sulph., gr. j.; acid sulph. aro., 9, s.; syrup rhei. comp. ℥j.; a teaspoonful every two hours. Dr. Vincenz, of Millstadt, St. Clair County, reports that the months of August, September, and October constitute their sickly season.

That the diseases are intermittent or remittent fevers, with more or less of bilious symptoms; that happily they are very amenable to the following treatment:—Calomel, gr. x.; vel ℈j., and rhubarb or jalap the same quantities; followed, if necessary, by castor oil, frequent sponging with cold water, till the dry, hot skin gets cool, soft, and moist. In the morning, at two or three o'clock, he gives quinine, gr. vi., and repeats every two hours till three doses are taken. He prefers giving the quinine thus early in the morning, because that usually, even in continued fevers, there is some abatement of febrile symptoms at that time; but, nevertheless, considers it perfectly safe to give the quinine at any time, if desirable, *provided* cold water is at the same time applied liberally both externally and internally. In some cases he prefers administering an emetic before the purgative powder. He remarks also upon the liability to relapses, upon any fatigue or indiscretion, about the tenth or twelfth days; and in such cases he gives from 6 to 9 grs. quinine at bedtime, a week after the fever has been broken, and repeats it at intervals of a week for three or four times. He also confirms the opinion, that a greater number of cases of periodic fevers occurred last year than he has known during a residence in Millstadt of eleven years. Connected with the unusual prevalence of periodic diseases last year, we had anticipated much interesting information and instructive details from our correspondent at Vandalia (Dr. F. B. Haller); but he briefly states: "The topography of our county is such as to indicate the diseases usually occurring in it, having the Okaw river extending through the centre, with a bottom, on an average, two miles wide, filled with lakes and lagoons, and always subject to inundation whenever we have a freshet; this produces bilious, remittent, intermittent, and congestive fevers, the most usual of which occur throughout the whole year, and to a great extent modifies all our diseases, and are the prevailing diseases through the summer and autumn months. They are much more common when we have a wet summer, and a dry, warm fall, and pretty much subside as cold weather sets in."

From Dr. H. R. Payne, of Marshall, Clark County, we have received a valuable paper, from which we shall levy considerable contributions. He says:—

"The most prevalent diseases we have, are those of a malarious origin, intermitting and remitting fevers. This, however, needs some qualification. The situation of Marshall is on an elevated ridge of what is called 'barren land,' on the east of us flows a stream some three miles distance, and the soil of the same character as the Wabash bottoms. This stream empties into the Wabash river east of us at its nearest approach to us. On our west, at a distance of three miles, flows another stream, the bottoms of which are also subject to overflow. Up to the last season, the persons living on the barren lands have enjoyed comparative immunity from intermittent or remittent fevers, the sickness being chiefly confined to the bottom lands by the streams just alluded to and to the Wabash bottom. Last season, however, the order of things was reversed. There was comparative immunity in the bottoms, but almost universal sickness on the barrens. We suffered probably less at Marshall than at any other point: both north and south of us nearly *every* family was attacked, and in many instances every member was prostrated. I ascribe this to the fact, that our town is situated on an elevated point; there being a gradual inclination in each direction the water was soon carried off, whilst in the other localities, possessing the same character of soil, the land was flat, allowing the water to stand and stagnate. The sickness broke out in the latter part of July, and continued without much cessation until the middle of November. The sickness I ascribe to the heavy rains, commencing in the early part of the spring season and continuing more or less up to the month of July: an unusual amount of vegetation sprung up, which readily decomposed, the heavy rains being followed by very warm, sultry days. The symptoms and cause varied but little from the symptoms of remitting and intermitting fever as they usually occur. The prognosis was in all cases favorable, with the exception of those cases complicated with typhoid symptoms; these complications occurring later in the season. The complication of typhoid symptoms made the disease much more formidable; and where it occurred in persons of a broken-down or debilitated constitution, made it still more unfavorable, proving fatal in several instances. One case, I have in view, was attacked with intermitting fever about the 10th October: he regarded it as nothing serious, and hence paid but little attention to it, probably took a dose of purgative medicine. In about four days from the commencement of the intermitting fever, typhoid or nervous symptoms developed themselves, which proved fatal in some seven days. His constitution was much shattered by previous sickness; but I am well satisfied, had quinine and blue mass been given at the proper time, they would entirely have prevented the development of typhoid symptoms. After the disease had assumed this form, the following were the characteristic symptoms: tongue dry and red at tip and edges, with a yellow bilious coating closely adherent in the centre; pulse 130 at its height, easily compressed, but followed by slight remissions in the morning; delirium greatest during the exacerbation of the fever; great susceptibility to the action of purgative medicines, even blue mass, without it was combined with opium, was followed in four or five hours by copious offensive watery stools; subsultus tendinum commenced early and became violent as the disease advanced; hardness of hearing was prominent; late in the disease great distention of the bowels; epistaxis presented itself early in the disease before the typhoid symptoms set in. The patient sank into a stupor from which he could not be aroused, and died on the morning of the eighth day from the time the typhoid symptoms first presented themselves."

A number of other cases, in the hands of different practitioners, proved fatal with the same symptoms.

"The prognosis in such cases was always unfavorable; but there were a number of cases, occurring in more vigorous persons, the majority recovering. The proportion of deaths, including all these cases, from what I can learn, is about 1 in every 6 or 7. Of the purely *bilious* forms of fever, there were no deaths; these cases were mostly arrested in from three to seven days by the use of calomel or blue mass, followed by quinine during the intermission. Those cases complicated with *decided* typhoid symptoms, required a different treatment. Calomel was given in smaller doses and combined with opium; quinine was also administered more for its *tonic* effect, as it utterly failed in *every* case in which it was given to break up or cut short the disease. The treatment was upon the whole expectant, varying but little from the course recommended by Dr. Wood in his article on Enteric or Typhoid Fever. Of the congestive form of fever there occurred in my practice several cases; all of these recovered: the basis of treatment was calomel and quinine; external applications, such as mustard to the spine and extremities, were used to restore reaction, and, where required, internal stimulants were administered.

Dr. McBane, of Metropolis City, in a very brief letter, (and from a man of his experience we ought to have received much valuable information,) states, that chill and fever, ague, &c., usually prevail in the marshy districts during the last four fall months, but that in the past season they continued till March last, and are rarely fatal. That, "after all extreme changes from hot to cold, we have many cases of congestive chills and fevers, often terminating in typhoid pneumonia, if not fatally." Of the treatment, or the special symptoms, he says nothing.

From Dr. Nance, of Lafayette, Stark County, we have received a very copious and valuable paper, which we append to this report. He states, that in his neighborhood, (refer to the topography in his letter,) during the latter part of summer and all autumn, miasmatic diseases prevail most extensively, consisting of intermittents and remittents, and some cases of congestive fever, and pernicious intermittents. That he considers the character of the previous weather determines the class of sickness; that frequent showers, followed by hot sunshine, if the weather has been previously dry, seem to develop periodic disorder; and that he is confident that these diseases have not prevailed in the same ratio since 1846, when the type was very analagous. That in both years he adopted pretty much the same treatment, to-wit: mercury and quinine, with the difference that this year he also gave a fair trial to the *Sulphate of Cinchonæ*, and is satisfied that it possesses many, if not equal advantages with the quinine. That, in 1855, complications but rarely occurred; while, in 1854, nearly all intermittents were complicated with diarrhœa. The Doctor does not say what was the usual condition of the alvine discharges during the past season, but he remarks that the prognosis was usually favorable and no sequelæ; but that relapses were frequent, without a continuation for 14 or 21 days of the quinine or the cinchonæ, and even then an occasional relapse occurred. We like the plan of Dr. Vincenz better. But it might, we think, fairly be asked, were these returns of the same disease always to be considered as relapses, seeing that the individuals were all the time exposed to the same causes which produced the first attack? We confess we doubt it.

The only information in regard to periodical fevers in Chicago, during the past season, we find in Prof. Davis' very able and valuable report to the Cook County Medical Society, in October last, upon the Sanitary Characteristics of Chicago. Therein he remarks, "That attacks of ordinary intermittent and remittent fevers have been more frequent during the month of September than for several years past." We suppose, therefore, Chicago, to a great extent, escaped the general endemic influence.

Under this head, we presume, should also be noticed an article in the *North-Western Journal* for January, 1856, by A. E. Goodwin, M.D., of Rockford, Ill., upon Intermittents and their Remedies; and while, with the editors of that journal, we are pleased to see attention directed to our indigenous medical botany; and while we fully coincide in the author's detestation of hobbyism, we confess we cannot hear our old friend Quinine so disrespectfully treated without speaking up in his defence. For ourselves, we are bound to say, that he is a good and trusty friend who never fails to do good service whenever he is applied to under proper circumstances. That it would be very desirable to find some other friends as good and trustworthy, we cheerfully admit: but to say, that, so far as has yet been proved, "There are *equally as good* and *better* remedies than cinchona or its alkaloids, for fever and ague;" "that it is not the specific par excellence; that we have indigenous remedies far preferable as a curative, as feasible preparations, and at less expense;" "that quinine will stop the chill, but that other remedies are more certain and reliable:"—to such statements we must beg to demur, and to express our fear that Dr. Goodwin in avoiding one hobby has mounted another, and one that looks very much as if it had been kept in the stable of an eclectic steamer. As regards the articles enumerated in his paper, we presume there are few of the profession in the West but who are acquainted with them, and who, while admitting their tonic properties, and in some degree their antiperiodic powers, are fully aware of one great objection to their use, viz.: the bulk such preparations occupy, and the difficulty therefore in many cases of administering them, where the patient is fastidious or where nausea exists, besides the uncertainty of the strength of any given parcel. As regards salacine, we have, during the past summer, tried it in several cases, but were greatly disappointed, while the large quantity required rendered its administration inconvenient. The decoction of hickory, willow, and dogwood barks, and sometimes even with the addition of whisky, (not having the fear of our friend Dr. Davis before our eyes,) has been a common recommendation of the Chairman of this Committee for many years past, as a prophylactic in the fall season, for persons living on low overflowed lands, or in their vicinity. As regards another remedy, to which attention has only recently been much directed, viz., the sulph. cinchonæ, we are pleased to be able to confirm the testimony of Dr. Nance, of Lafayette, already quoted. We tried it, during the past fall and spring, in several cases, with successful results; and though our experience, so far, would not lead us to ascribe to it the same power as an antiperiodic, or the same sedative effects upon the vascular system that quinine possesses, yet it has the advantage of not producing such unpleasant cerebral sensations, is cheaper, and, therefore, where a tonic of this class is required, especially as a prophylactic, we consider it deserving of preference. We have generally administered it according to the following formula, derived from a paper in the *American Journal of Medical Sciences*, for January, 1856, page 269 to 275; it is the same used at the Philadelphia Dispensary, by Dr. George Martin, and is as follows:—Sulph. cinchonæ, grs. xxxij.; tinct. ferri murf, ℥ ss.; aquæ, ℥ iv.; to this we added about 10 drops muriatic acid, and generally 1 grain muriate of morphia, directing a large tablespoonful to be given in water three times a day to an adult—the combination of the chalybeate doubtless adding greatly to its virtues when used as a preventive of relapse.

And now, in regard to the statistics and experience of the past season in Periodic Diseases, your committee have access to but one other source, viz., the Chairman's own notes: these for simplicity's sake have been tabulated. They begin on 1st Jan., 1855, and to avoid fresh numbering throughout we will take them from the beginning, as indeed they include only 11 cases of periodic disease prior to 7th June last. We proposed at first to have copied into this report the symptoms and treatment of each case, but finding that would have doubled the bulk of what is anyhow a voluminous document, we have merely noted the prominent peculiar symptoms; and the general tenor of treatment alone is given in a few lines at the end. The object of constructing this table was to present for future reference a fair picture of the ages and sex of those attacked, with the type of disease and its tendencies, the liability to relapse, and the results of treatment. Could we also have supplied, as Dr. Davis has done, accurate meteorological records to accompany it, its value we think would have been considerable. It is rarely such an opportunity presents for observing this class of diseases as was presented last year; and though not generally regarded as a grave disease, we all know how soon, by neglect or mismanagement, it may pass into one of the most fatal destroyers of human life. To place, therefore, on file the statistics of its operations in a large number of cases, we have thought desirable. These tables comprise 356 cases of intermittent, remittent, congestive, and pernicious fevers, treated between 1st Jan. 1855, and 17th May, 1856. We would remark, that, in some few cases, the ages have been presumed from personal knowledge, but cannot be far from the truth. In some other cases, the type and the sex were omitted in the notes: these cases must go for what they are worth. Three patients died:—cases 53 and 153 succumbed the night after prescription; the first would not take *any* medicine; the other was dead when the messenger got back with the medicine —case 111 died in two days after first seen; she had been sick many days under the tender mercies of quacks.

In some of the systematic writers (see *Cyclopædia of Practical Medicine*, Art. Intermittent Fever,) it is stated, that the paroxysm of quotidian is early in the morning. These tables will show the error of this opinion. Again—it is the opinion of my colleague, Dr. Harris, and of many others, "that our fevers gradually assume a graver type from September to January,"

but that they *all* belong to the Periodic family. Will these tedious tables support this view? The gravest cases and the most protracted, with one or two exceptions, occurred in September and October; whilst in November, December, and January, &c., the cases were mostly simple intermittents—relapses in many cases, and soon dismissed. Another fact is presented, that sometimes, for many days, all the cases would be of one type and then change, nearly every case for some days after presenting another type. Occasionally, chiefly females were attacked; again, at another time, it was males that were the sufferers. We do not attempt to speculate on the causes of these diversities—till we extracted the cases into these tables, we had not recognized these facts ourselves.

| No. | Date, 1855. | Age. | Sex. | Type. | Remarks. |
|---|---|---|---|---|---|
| 1 | Jan. 1, | 3 | male | tertian | chill in morning. |
| 2 | 13 | 3 | | quotidian | remittent at 4 P.M., costive. |
| 3 | 16 | 3 | female | " | " delirium during fever, costive. |
| 4 | 16 | 9 | female | tertian | intermittent, relapse was quotidian. Tongue red at edges. |
| 5 | 17, 18 | 3 | female | irregular | remittent, fever all the time, skin hot, face flushed, pulse 140. |
| 6 | Jan. 28, and Feb. 5 | 26 | female | irregular | had remittent fever all last summer; is anœmic, anasarcous, urine scanty, has cough, is faint and chilly, costive. |
| 7 | March 24 | 35 | female | quotidian | sun pain. |
| 8 | April 2 | 60 | " | " | chills, with catarrh. |
| 9 | 9 | 24 | " | d'uble tertian | tremblings and pains in bones twice in 24 hours. |
| 10 | 9 | 30 | " | quotidian | chills. |
| 11 | May 26, 29, & June 28, & Aug. 18. | 16 mo. | male | " | chills, with dysentery, which during its several relapses became a white diarrhœa. |
| 12 | June 7 | 60 | female | quotidian | chill, intense pain in right side of abdomen, with syncope. |
| 13 | June 6, 11 | 2½ | female | quotidian | chills, diarrhœa. |
| 14 | July 2 | 30 | " | " | had chills for months, been under quacks, is asthmatic and anœmic. |
| 15 | 5, 6, 8 | 60 | female | quotidian | remittent, with great intestinal irritation and prostration, costive. |
| 16 | 5, 6, 8 | 62 | male | tertian | intermittent, breath fetid, quacking. |

| No. | Date, 1855. | Age. | Sex. | Type. | Remarks. |
|---|---|---|---|---|---|
| 17 | July 10 | 25 | female | quotidian | remittent, diarrhœa, stools lead-colored, has a congestive tendency, anœmic. |
| 18 | 12 | 55 | female | quotidian | remittent, habitually costive. |
| 19 | 16, 17 | 4 mo. | female | " | intermittent, green diarrhœa. |
| 20 | 17 | 35 | male | " | " |
| 21 | 20 | 16 mo. | " | " | " vomiting, diarrhœa. |
| 22 | 19 | 4 | female | tertian | remittent |
| 23 | 20, 22 | 25 | male | " | intermittent |
| 24 | 21 | 4 | female | quotidian | remittent, cold clammy sweats, hemorrhage from bowels and nose, diarrhœa. |
| 25 | 23 | 84 | female | quotidian | intermittent, congestive epilepsy during chill. |
| 26 | 27 | 14 | male | tertian | remittent. |
| 27 | 30, and Aug. 4, 5 | 48 | " | quotidian | remittent, with neuralgia of skin, and sciatic nerve. |
| 28 | July 30 | 18 | female | tertian | intermittent. |
| 29 | July 30, and Aug. 1, 8 | 45 | " | " | remittent. |
| 30 | July 31 | 50 | male | " | " congestive tendency. |
| 31 | Aug. 2, 5 | 40 | " | " | " chills aggravating. |
| 32 | Aug. 2, 3, 4, 5, 6, 7, 8, 9, 10, 12 | 40 | " | quotidian | intermittent, pernicious, hemorrhage from bowels. |
| 33 | Aug. 2, 4 | 12 | | quotidian | intermittent. |
| 34 | 3, 5, 8, 9 | 18 mo. | female | " | " diarrhœa. |
| 35 | 3, 4 | 4 | " | | remittent, costive. |
| 36 | 4, 5, 6, 7 | 35 | male | " | intermittent; chill, fever, and sweat all at once; vomits bile. |
| 37 | Aug. 4, 5, 6, 7, 8, 9, 10, 11, | 10 | male | | intermittent, general anasarca. |
| 38 | Aug. 4, 5 | 5 | male | quotidian | intermittent, congestive stupor. |
| 39 | Aug. 7,8,9,10 | 9 | " | " | intermittent, diarrhœa. |
| 40 | Aug. 3, 4, 5, 6, 9 | 12 | " | " | remittent, costive. |
| 41 | Aug. 5 | 45 | female | quotidian | intermittent, pregnant. |
| 42 | 6 | 25 | " | tertian | " relapse 17. |
| 43 | 6 | 2 | " | " | " diarrhœa. |
| 44 | 6 | 25 | " | " | " |
| 45 | 6, 7, 8 | 55 | " | quotidian | intermittent, chills protracted, fever and sweat slight, pain in hypogastrium. |
| 46 | 7 | 32 | male | quotidian | remittent, shiverings alternating with fever all day. |
| 47 | 7, 16 | 2 wks. | | " | remittent, cholera infantum. |
| 48 | 8 | 20 | male | " | remittent. |
| 49 | 9, 10, 12 | 25 | " | " | intermittent, chills very protracted and anticipating fever very high, very little sweat, vomiting. |
| 50 | 9, 10, 12 | 24 | female | quotidian. | intermittent " |
| 51 | 9, 10, 12 | 4 | " | " | " " |
| 52 | 9 | 4 | male | " | " cholera infantum. |

| No. | Date, 1855. | Age. | Sex. | Type. | Remarks. |
|---|---|---|---|---|---|
| 53 | Aug. 12 | 80 | male | quotidian. | intermittent, sick a week, this morning chill, fever and sweat all at once, dysentery, intense abdominal pain, *died at night.* |
| 54 | Aug. 12, 13, 15, 17 | 50 | male | tertian | intermittent, but after first chill they became indistinct; fever gradually came on about 4 P.M., pain in abdomen; stools like cholera, mixed with blood; intense pain in head. |
| 55 | Aug. 13, 15 | 40 | female | quotidian | intermittent, costive, icterode, vomiting. |
| 56 | 13 | 21 | " | " | remittent, choleraic symptoms. |
| 57 | 13 | 20 | " | " | intermittent, faintness during chills. |
| 58 | Aug. 14, 15, 16, 17 | 10 | " | " | congestive remittent, skin hot and sweaty, shivering and high fever; stools dark, with much venous blood. |
| 59 | 15 | 24 | female | quotidian | remittent, shivering and high fever. |
| 60 | 15 | 25 | " | " | remittent, shivering and high fever, vertigo. |
| 61 | 15 | 28 | male | " | remittent, shivering and high fever. |
| 62 | 16, 19 | 60 | female | tertian | intermittent, intense pain in toes during chill. |
| 63 | 16, 19 | 20 | " | quotidian | remittent, diarrhœa, hands cold all the time. |
| 64 | 16 | 28 | male | tertian | intermittent. |
| 65 | 18, 19 | 3 | " | " | " diarrhœa. |
| 66 | 18 | 45 | " | quotidian | " |
| 67 | 22 | 40 | " | irregular | remittent. |
| 68 | Aug. 22, 23, 24, 25, 26 | 54 | female | | first chill on 21st, fever and pain in stomach continues. |
| 69 | Aug. 31 and Sep. 6, 13, and Oct. 23 | 26 | " | tertian | intermittent, got wet on back in a shower. Paralysis of right side came on, been so about a month. |
| 70 | Aug. 31 and Sep. 1, 2, 3 | 45 | male | quotidian | remittent, has chills first, then fever gets more continuous, abdominal pulsations, intense frontal headache, and pain in hypochondria. |
| 71 | Aug. 31 and Sep. 1, 2 | 30 | male | quotidian | remittent, skin hot and sweaty, but is shivering, bowels torpid, pulse very small and weak. |
| 72 | Aug. 31 and Sep. 1, 2, 17, 18, 20 | 5 | male | quotidian | intermittent chill at noon, with convulsions; has enormous head. |
| 73 | Aug. 31, Sep. 1, 2, 3, 22, 24 | 4 | female | quotidian | intermittent, costive. |
| 74 | Aug. 31, Sep. 1,2,3,22,24 | 4 | female | quotidian | intermittent, diarrhœa. |

| No. | Date, 1855. | Age. | Sex. | Type. | Remarks. |
|---|---|---|---|---|---|
| 75 | Sep. 4 | 40 | female | quotidian | intermittent, colicly diarrhœa with blood, been sick two weeks. |
| 76 | Sep. 5, 6, 7, 8, 10, 11, 13, Oct. 21 | 40 | female | quotidian | intermittent, pernicious; chill of morning, becomes numb, cold, speechless, almost pulseless, then fever and sweat come together. Is suckling. |
| 77 | Sep. 6 | | male | | chills, fever, headache. |
| 78 | 6 | | female | | " " " |
| 79 | 6, 7, 8 | 18 | " | quotidian | remittent; fever, and sweat, and shivering all the time; small weak pulse, and faintness. |
| 80 | Sep. 7, 8, 10 | 28 | female | quotidian | remittent, fever and diarrhœa. |
| 81 | 9, 11, 12 | 48 | male | " | remittent, fever all the time, sweat and chilly. |
| 82 | 9, 11, 12 | 8 | " | 1st chill | cold nearly all the time, stupor, stools like milksick. |
| 83 | 9, 11 | 6 mo. | female | quotidian | intermittent, chills anticipating. |
| 84 | 9, 11 | 25 | " | tertian | intemittent, *relapse of* 42. |
| 85 | 8, 9, 10 | 17 | " | " | " becoming remittent. |
| 86 | 10 | 32 | " | quotidian | remittent, stools black, quackery. |
| 87 | 11 | 10 | male | " | intermittent. |
| 88 | 11 | 8 | " | " | remittent. |
| 89 | 12 | 45 | " | " | intermittent. |
| 90 | 14, 20 | 68 | " | " | " |
| 91 | 14, 17 | 14 | female | " | " |
| 92 | 15 | 15 | male | " | " has been tertian. |
| 93 | 16 | 9 | female | tertian | " |
| 94 | Sep. 16, 17, 18, 20 | 37 | male | quotidian | remittent, fever aggravating; stools dark, offensive, like milksick. |
| 95 | Sep. 16, 17, 18, 20 | 28 | female | quotidian | remittent, fever aggravating; stools dark, offensive, like milksick. |
| 96 | Sep. 17, 18, 20, 25, 26 | 17 | male | 1st chill | remittent, shiverings, headache, sweats, fever, and bloody diarahœa. |
| 97 | Sep. 25, 26 | 45 | male | 1st chill | remittent, shiverings, headache, sweats, fever, and bloody diarrhœa. |
| 98 | 17, 18, 20 | 21 | " | tertian | remittent, fever, headache, diarrhœa, sick a week. |
| 99 | 18 | 25 | " | quotidian | remittent, shiverings, pain in stomach and bones. |
| 100 | 19 | | child | | remittent, fever and sweats, chill not noticed. |
| 101 | 19 | | " | | remittent, fever and sweats, chill not noticed. |
| 102 | 19 | 44 | male | irregular. | remittent, at first no recognized chill, but had pain in left shoulder at certain hour, then fever, stools black and offensive, been sick 6 weeks. |

| No. | Date, 1855. | Age. | Sex. | Type. | Remarks. |
|---|---|---|---|---|---|
| 103 | Sep. 19 | 32 | female | tertian | remittent. |
| 104 | 19 | 8 | male | " | intermittent. |
| 105 | 19 | 40 | " | quotidian | remittent, shiverings, fever and sweats, intense headache, stools black. |
| 106 | 19 | 12 | male | quotidian | remittent, shiverings, fever and sweats, intense headache, stools black, anœmic. |
| 107 | 21 | 42 | female | quotidian | intermittent. |
| 108 | 21, 22 | 2 wks. | male | " | remittent. |
| 109 | 21, 22 | 8 | female | " | remittent, intense headache, then fever, hot and dry skin, hands and feet cold all the time, stools dark. |
| 110 | 21, 22 | 6 | female | quotidian | remittent, intense fever, flushed face, chill, heavy sleep, dark scanty stools. |
| 111 | Sep. 22,23,25 | 56 | female | | sick two weeks under quacks, taken with headache, chills, fever; now skin not very hot, has bloody involuntary diarrhœa, gangrene of skin on chest from blister. |
| 112 | 23 | 15 | male | | chill and high fever. |
| 113 | 23 | 20 | " | quotidian | remittent, sick a week, taken with headache, no chill observed, bowels open. |
| 114 | 23 | 14 | male | quotidian | intermittent. |
| 115 | 23 | 20 | female | " | " |
| 116 | 23 | 2 | " | " | " |
| 117 | 24 | 25 | " | tertian | " |
| 118 | 24, 26 | 48 | male | quotidian | remittent, headache, shiverings, vomiting, costive. |
| 119 | 24, 26 | 45 | female | quotidian | remittent, neuralgia of right leg and thigh, worst at 2 P.M. |
| 120 | 24 | 10 | male | quotidian | intermittent, becoming remittent. |
| 121 | 24 | 46 | " | tertian | intermittent. |
| 122 | 24 | 17 | " | " | " |
| 123 | Sep. 24, 25, 26, 27, 28, 29, 30 | 7 | " | irregular | remittent, sick a week, quacked; taken with headache and high fever, at times hands, arms, legs, and feet cold for hours. |
| 124 | Sep. 24, 25 | 35 | male | quotidian | remittent. |
| 125 | 25 | 18 | " | tertian | intermittent, been quacking. |
| 126 | 25 | 14 | " | quotidian | intermittent, been quacking. |
| 127 | 26 | 16 | female | " | intermittent. |
| 128 | Sep. 26, 27, 28, 29, 30, Oct. 2, 5, 9, 10, 13, 14, 15, 17, 18, 19, 20 | 30 | male | irregular | remittent, under quack for 12 days, has diarrhœa, dysphagia, fever, stupor, sleeplessness. |
| 129 | Sep. 28 | 48 | male | quotidian | intermittent. |
| 130 | 28 | 15 | female | " | " |
| 131 | 28 | 12 | " | tertian | " |

| No. | Date, 1855. | Age. | Sex. | Type. | Remarks. |
|---|---|---|---|---|---|
| 132 | Sep. 28,29,30 | 40 | female | quotidian | remittent, aborted week before, flooding returns with each chill. |
| 133 | Sep. 28,29,30 | 12 | male | tertian | intermittent. |
| 134 | 28,29,30 | 10 | " | " | remittent, chills obscure, incoherence. |
| 135 | Sep. 29, 30, and Oct. 1, 2 | 60 | female | " | intermittent, pernicious, giddiness, incoherence. |
| 136 | Sep. 30 and Oct. 1 | | boy | tertian | intermittent. |
| 137 | Sep. 30, 31 | 26 | male | began quotidian | remittent, now irregular, shivering, fever, bronchitis, costive. |
| 138 | 30 | 14 | " | quotidian | remittent. |
| 139 | Sep. 30 and Oct. 1 | 21 | female | tertian | remittent, fever, restlessness, vomits blood. |
| 140 | Sep. 30 | 20 | male | tertian | intermittent. |
| 141 | 30 | 22 | female | " | " |
| 142 | Oct. 1 | 5 | " | quotidian | " |
| 143 | 1 | 12 | male | " | " costive. |
| 144 | 1 | 8 | " | " | " " |
| 145 | 1, 2, 4 | 28 | female | " | shiverings, achings in back and limbs, costive. |
| 146 | 1, 2, | 42 | " | " | chills, vomiting, diarrhœa. |
| 147 | Oct. 3, 12, 13, 15 | 4½ | " | " | intermittent, and on 12th epistaxis. |
| 148 | Oct. 3 | 48 | " | " | remittent, costive. |
| 149 | 3 | 8 | " | " | intermittent, costive. |
| 150 | 3, 4 | 34 | male | " | remittent, 3d chill, fever very high, no sweat. |
| 151 | 3, 4 | 29 | female | tertian | chills, fever, diarrhœa. |
| 152 | 3, 4 | 8 | male | " | chills, fever, face flushed. |
| 153 | 3 | 12 | " | quotidian | intermittent, congestive, high fever, hot sweat, had fit and died in 3d chill. |
| 154 | 4 | 10 wks | | quotidian | intermittent. |
| 155 | 4 | 6 | female | | chills, vomits. |
| 156 | 4, 5 | 40 | male | | remittent, shiverings, fever, headache, pulse very small. |
| 157 | 5, 6, 7 | 46 | female | tertian | intermittent, 3d chill lasted 2½ hours, great epigastric oppression, retching, dysuria and dyspnœa, pulse small, skin cold. |
| 158 | Oct. 5, 6, 7, 8, 19 | 45 | male | quotidian | remittent, pulse small, costive. |
| 159 | Oct. 6 | 6 | female | " | chills, costive. |
| 160 | Oct. 6, 7, 9, 11, 16 | 36 | male | " | remittent; fever, sweat, and chill mingled; costive. |
| 161 | Oct. 6, 7, 9, 11 | 25 | female | " | sick since 3d, pulse weak, remittent. |
| 162 | Oct. 6 | 12 | male | " | |
| 163 | 7, 9 | 40 | female | | remittent, gastric oppression, dyspnœa. |
| 164 | 7, 9 | 59 | male | 1st chill | on 7th, 8 A.M., *lasted all day.* |
| 165 | 8 | 20 | " | | chills. |

| No. | Date, 1855. | Age. | Sex. | Type. | Remarks. |
|---|---|---|---|---|---|
| 166 | Oct. 8 | 25 | male | tertian | intermittent, great gastric oppression during chill. |
| 167 | 8 | 33 | " | | remittent, nausea. |
| 168 | 8, 10 | 18 | " | tertian | intermittent, chill anticipating |
| 169 | 8, 10 | 24 | female | " | |
| 170 | 9 | 12 | male | quotidian | remittent, belly tender, diarhœa. |
| 171 | 9 | 36 | female | tertian | intermittent, menorrhagia with the chill. |
| 172 | 10 | 12 | " | | chills. |
| 173 | Oct. 10, 11, 12, 13, 14, 16 | 35 | male | quotidian | remittent, 1st chill on 6th, diarrhœa, great irritation of bladder. |
| 174 | Oct. 10, 11, 12, 13, 14, 16 | 33 | female | | remittent, nausea, faintness. |
| 175 | Oct. 10, 11, 14 | 40 | male | quotidian | remittent, 1st chill on 8th, chill, fever, and sweat blended. |
| 176 | Oct. 10, 11 | 46 | " | tertian | intermittent, profuse *epistaxis.* |
| 177 | 10 | 31 | " | " | remittent. |
| 178 | Oct. 11, 12, 14, 16 | 49 | " | quotidian | remittent, shiverings, fever, sweats. |
| 179 | Oct. 11, 12, 14, 28 | 14 | female | tertian | intermittent, costive. |
| 180 | Oct. 11, 12, 13, 14, 15, 19, 20 | 54 | " | quotidian | remittent, 1st chill on 7th, no sweat. |
| 181 | Oct. 11, 12, 13, 14, 15, 19, 20 | 24 | male | quotidian | intermittent, had 7 chills, anticipating. |
| 182 | Oct. 12 | 29 | male | tertian | intermittent. |
| 183 | 12 | 34 | female | " | " costive. |
| 184 | 12 | 10 | male | " | " diarrhœa. |
| 185 | 12 | 9 | female | " | " |
| 186 | Oct. 13, 14, 15, 16, 17, 18, 20 | 43 | " | quotidian *pernicious* | intermittent; had chills, aggravating for some time, on 12th chill lasted from 8 A.M. till 5 P.M.; vomiting and purging all the time, urine scanty, reaction very imperfect and accompanied with gluey sweat, stupor all the time, great dyspnœa. |
| 187 | Oct. 14, and Nov. 23, Dec. 7 | 23 | male | tertian | intermittent. |
| 188 | Oct. 14, 16, 17, 28 | 52 | " | quotidian | intermittent, 1st chill on 8th, lay in stupor for 2 days, on on 28th relapse from overexertion, with chill, stupor, vomiting. |
| 189 | Oct. 14, 15, 17 | 51 | male | quotidian | remittent, profuse diarrhœa. |
| 190 | Oct. 14, 15, 17 | 48 | female | double quotidian | intermittent, intense headache, nausea. |
| 191 | Oct. 14, 15, 17 | 7 | " | quotidian | intermittent, chills aggravating. |

| No. | Date, 1855. | Age. | Sex. | Type. | Remarks. |
|---|---|---|---|---|---|
| 192 | Oct. 14, 15, 17 | 14 | female | 1st chill | |
| 193 | Oct. 15, 17, | 3 | " | quotidian | intermittent. |
| 194 | 16, 27 | 27 | " | tertian | remittent. |
| 195 | 16 | 16 | " | " | remittent. |
| 196 | 16 | 24 | " | quotidian | intermittent, chills aggravat'g |
| 197 | 16 | 6 | male | " | intermittent. |
| 198 | 16 | 34 | " | " | remittent. |
| 199 | Oct. 16, and Nov. 19 | 26 | female | " | remittent. |
| 200 | Oct. 16 | 15 | " | " | intermittent. |
| 201 | 16, 17 | 28 | " | " | remittent, costive. |
| 202 | 16 | 26 | male | " | intermittent. |
| 203 | 16 | 19 | female | " | intermittent. |
| 204 | 17, 18 | 5 | " | " | intermittent, vomiting. |
| 205 | 17 | 3 | male | tertian | intermittent. |
| 206 | 17 | 12 | " | " | intermittent. |
| 207 | 17, 19 | 7 | female | quotidian | remittent. |
| 208 | 17, 19 | 4 | " | " | intermittent. |
| 209 | 17, 19 | 9½ | male | " | intermittent. |
| 210 | 17 | 6 | | double tertian | intermittent, 11 A.M. one day, 2 P.M. other. |
| 211 | 17, 20 | 60 | female | | remittent, vertigo, vomiting, been sick a month under quack. |
| 212 | Oct. 18, 19, 21, 24 | 24 | male | quotidian | remittent, sick a month, 1st chill on 7th, diarrhœa with blood, vomits blood. |
| 213 | Oct. 18, 19, 21, 22, 23, 24, 25 | 40 | female | | remittent, had chill on 12th, under quacks. |
| 214 | Oct. 18, 20 | 18 | male | quotidian | remittent, diarrhœa, vomiting, relapse. |
| 215 | 18 | 25 | male | quotidian | intermittent, chill anticipating three hours. |
| 216 | 19 | 4 | female | quotidian | intermittent, relapse. |
| 217 | 19 | 10 | male | " | intermittent. |
| 218 | 20 | 36 | " | chill 3 weeks ago | remittent, constant pain in head, been quacking. |
| 219 | 20 | 34 | male | quotidian | intermittent. |
| 220 | 20, 22 | 29 | female | " | remittent, (confined 3 weeks ago,) chills aggravating, hysterical, anœmic. |
| 221 | 20, 22 | 5 | female | quotidian | intermittent. |
| 222 | 20, 22 | 2 | male | " | intermittent. |
| 223 | 20, 21 | 3 | " | " | remittent, stupor, sick several weeks. |
| 224 | 21 | 5 | female | | intermittent, been salivated. |
| 225 | 21 | 3 | male | | remittent, stupor, fever, achings. |
| 226 | 22, 21 | 40 | female | | remittent slight, diarrhœa. Relapse 76. |
| 227 | 21 | 12 | " | quotidian | intermittent. |
| 228 | Oct. 20, 23, 24, 25, 26, 27, 28, 29, 30 | 17 | male | " | remittent, great gastric irritation. |

| No. | Date, 1855. | Age. | Sex. | Type. | Remarks. |
|---|---|---|---|---|---|
| 229 | Oct. 22 | 3 wks. | | | intermittent. |
| 230 | 23 | 20 | female | quotidian | " |
| 231 | 23 | 6 mo. | | " | " |
| 232 | 23 | 50 | " | " | " chill at night. |
| 233 | 24 | 27 | male | | " |
| 234 | Oct. 24, 25, 26 | 40 | " | " | remittent, chill 10 A.M., intense headache, fever all the time, sweat with the fever. |
| 235 | Oct. 25, 26, 27, 28, 29, 30 | 10 | female | | remittent, great enteric irritation, in same house as 228. |
| 236 | Oct. 26, 27, 28, 29, 30 | 49 | male | quotidian | intermittent, stupor during fever, mental alienation during chill; on 29th, copious intestinal hemorrhage. Relapse, 178. |
| 237 | Oct. 26, 27, & Nov. 30 | 14 | female | quotidian | intermittent, relapse 179. |
| 238 | Oct. 27 and Nov. 3 | 24 | " | " | intermittent, diarrhœa and dysentery at daybreak each day. Relapse 181. |
| 239 | Oct. 27 | 24 | female | | intermittent, relapse 196. |
| 240 | 28 | 33 | male | quotidian | intermittent, chill 1 P.M. Relapse 167. |
| 241 | 28 | 28 | female | " | intermittent, chill at night. |
| 242 | Oct. 28, 29, 30, 31 | 23 | male | tertian | remittent, chills anticipating, great intestinal irritation and prostration. |
| 243 | Oct. 28, 29, 30, 31 | 15 | male | quotidian | remittent, sick in Chicago in July, now diarrhœa, pulse *very* weak. |
| 244 | Oct. 28, 29 | 40 | female | quotidian | remittent, chills anticipating, vomits, sleepless. |
| 245 | 28,29,30 | 25 | " | | remittent, diarrhœa, great prostration. |
| 246 | 29 | 13 | male | | remittent, was intermittent; 1st chill on 25th. |
| 247 | 30, 31 | 18 | " | tertian | intermittent. |
| 248 | Nov. 4 | 26 | " | " | intermittent. |
| 249 | Nov. 4, 5, 10 | 28 | " | | pernicious chill; pain commenced early on morning of 4th in right forefinger, extended up the arm and down the side and legs; *agony terrible;* skin cool; pulse 50, very weak and *dropping*. |
| 250 | Nov. 5 | 15 | female | irregular | intermittent. |
| 251 | 5 | 17 | " | quotidian | " chills anticipating. |
| 252 | 5 | 3 | | " | " 4th chill. |
| 253 | Nov. 5, 7, 8, 25 | 35 | male | " | remittent diarrhœa, vomits blood. |
| 254 | Nov. 6 | 5 | female | " | intermittent. |
| 255 | 6 | 19 | male | tertian | " |
| 256 | 6 | 8 mos. | | " | " |
| 257 | 6 | 28 | female | quotidian | remittent. |
| 258 | 7 | 3 | " | double quartan | intermittent. |
| 259 | 8 | 26 | " | tertian | " |

| No. | Date, 1855. | Age. | Sex. | Type. | Remarks. |
|---|---|---|---|---|---|
| 260 | Nov. 11 | 8 | | quotidian | remittent, was ague. |
| 261 | Nov. 13, 26, & Dec. 11 | 18 | male | tertian | intermittent, sick many weeks, quacking, chills delayed. |
| 262 | Nov. 13, 26, & Dec. 11 | 14 | " | quotidian | intermittent, sick many weeks, quacking, anœmic, costive. |
| 263 | Nov. 13, 15, 26 | 50 | " | irregular | intermittent, sick all summer under quacks. |
| 264 | Nov. 13 | 25 | female | | remittent, cough. Relapse 245. |
| 265 | 14 | 26 | " | tertian | intermittent, sick for months under quacks. |
| 266 | Nov. 15. 16, 17, 18 | 40 | " | | remittent, congestive, taken night of 14th with heaviness increasing to stupor, lays almost insensible, epigastric tenderness exquisite, pulse slow, costive, skin warm. |
| 267 | Nov. 17 | 24 | female | tertian. | intermittent, relapse 169. |
| 268 | 17 | 33 | male | quotidian | intermittent. |
| 269 | 17 | 15 | female | | intermittent, chills for 3 mos., costive. |
| 270 | Nov 18, 19, 20, 21, 22, 23, 25 | 49 | male | quotidian | remittent, stupor, passes urine involuntarily, incoherent; eyes wide open, stares, costive. Relapse 236. |
| 272 | Nov. 23 | 54 | female | quotidian | intermittent, diarrhœa. |
| 273 | 23 | 6 | " | | " |
| 274 | 24 | 7 | | | " |
| 275 | 24 | 35 | female | | " |
| 276 | 24 | 8 | | | " |
| 277 | 26 | 24 | " | quartan | " |
| 278 | Nov. 26, 27, 29, & Dec. 17 | 23 | male | quotidian | remittent, on 17th Dec. epistaxis. Relapse 242. |
| 279 | Nov. 29 | 9 | female | | intermittent. |
| 280 | 29 | 7 | " | | " |
| 281 | 29 | 4½ | " | quotidian | " cerebral symptoms. |
| 282 | 29 | 4½ | " | quotidian | intermittent. |
| 283 | 29 | 23 | male | | " |
| 284 | Dec. 3 | 29 | female | quotidian | " relapse 220. |
| 285 | 10 | 13 | male | tertian | intermittent; chill at first in morning, but got later and later, till they again began at 8 A.M. |
| 286 | Dec. 16 | 46 | male | quotidian | intermittent, fever high, sweat profuse. Relapse 176. |
| 287 | 18 | 35 | " | tertian | intermittent, urine scalds. Relapse 253. |
| 288 | 18 | 3 | female | quotidian | intermittent, chills at uncertain hours, constant desire to urinate. Relapse 258. |
| 289 | 18 | 29 | female | tertian | intermittent; no chills, but dragging pains in left side, every other day. Relapse 265. |

| No. | Date, 1855. | Age. | Sex. | Type. | Remarks. |
|---|---|---|---|---|---|
| 290 | Dec. 20, 22, 24 | 31 | male | tertian | intermittent for some weeks; got wet, and now has cough and spits blood while chill on. |
| 291 | Dec. 20, 22, 24, 25, 26, 28 | 35 | male | | remittent, costive, is consumptive, on 25th got pneumonia. |
| 292 | Dec. 24 | 4½ | female | quotidian | intermittent, relapse 281. |
| 293 | 25 | 37 | " | | " |
| 294 | 25 | 10 | | | " |
| 295 | 25 | 2 | | | " |
| 296 | Dec. 26, 28, 30 | 49 | male | quotidian. | intermittent, chill at night, convulsions with the chill. Relapse 270. |
| 297 | Dec. 28 | 23 | male | | intermittent, relapse 283. |
| 298 | 30 | 14 | female | | " " 237. |
| 299 | 30 | 20 | " | | " |
| 300 | 31 | 14 | male | | " relapse 262. |
| | **1856.** | | | | |
| 1 | Jan. 1, 4, 9 | 19 | male | irregular | intermittent, had chills for months, taken quinine, &c., in large quantities. |
| 2 | 5 | 4½ | female | quotidian | intermittent, relapse 292. |
| 3 | Jan. 13 and Feb. 2, 3, 9 | 23 | male | | intermittent, sick since 1st, slight chills, epistaxis. Relapse 278. |
| 4 | Jan. 23, 30, April 20 | 17 | male | tertian | intermittent. |
| 5 | Jan. 23 and April 14 | 23 | male | tertian | " relapse 297. |
| 6 | Jan. 23, 24 26, 27, 28, 29, 30, 31, Feb. 1 to 20, and 22, March 2, 5, 6, 8 | 17 | female | | remitting, chills slight, pulse 100, tongue clean, abdomen tender, diarrhœa, stools yellow, grainy water. *Qy.*—Typhoid? |
| 7 | Jan. 27, 28, 29, 30, and Feb. 2 | 15 | female | | remittent. |
| 8 | Feb. 1 | 3½ | female | quartan | intermittent, relapse 258. |
| 9 | 6 | 4½ | " | tertian | " relapse 281. |
| 10 | 6 | 4½ | " | " | " relapse 282. |
| 11 | 9 | 15 | " | quotidian | intermittent *at night*, amenorrhœa. |
| 12 | 9 | 13 mo. | male | tertian | intermittent, fever severe, diarrhœa, hands swollen and red. |
| 13 | 18 | 25 | " | quotidian | intermittent, costive. |
| 14 | Feb. 21, 25, 29 | 26 | " | " | intermittent, pleurodynia, sick a long time under quacks, anœmic. |
| 15 | Feb. 21, 26 | 50 | female | tertian | intermittent, sick all summer, ascites. |
| 16 | 23 | 80 | " | intermittent | intermittent, chills anticipating. |

| No. | Date, 1856. | Age. | Sex. | Type. | Remarks. |
|---|---|---|---|---|---|
| 17 | Feb. 25 | 48 | male | | intermittent. |
| 18 | Feb. 26 and March 8 | 49 | " | | intermittent, relapse 296. |
| 19 | Feb. 26 | 14½ | female | | " " 298. |
| 20 | Mar. 3 | 55 | " | quotidian | " chill 3 P.M. |
| 21 | 3, 25 | 5 | " | " | intermittent 8 A.M., costive, *on 25th tertian.* |
| 22 | 3, 25 | 5 | " | " | intermittent 5 A.M., diarrhœa, *on 25th tertian.* |
| 23 | 9, 7, 13 | 10 | " | " | intermittent, cough, costive. |
| 24 | 9 | 49 | male | | " |
| 25 | 11 | 9 | " | | " |
| 26 | 16 | 10 | female | | " |
| 27 | 17 | 6 | " | | " |
| 28 | 18, 22 | 5 | male | tertian | " |
| 29 | Mar. 19, Ap. 1 | 25 | female | irregular | intermittent, had chills for months, been under several doctors, always costive. |
| 30 | Mar. 19 | 7 | female | tertian | intermittent, periodic pains. |
| 31 | 21 | 19 | male | " | intermittent, delirium with the fever |
| 32 | 21 | 26 | " | | intermittent, relapse of 14, 1856. |
| 33 | 26 | 70 | " | tertian | intermittent. |
| 34 | 27 | 18 mo. | " | " | intermittent, anticipating. |
| 35 | 28 | 78 | " | " | intermittent, great dyspnœa during chill. |
| 36 | 30 | 37 | " | " | intermittent. |
| 37 | 30 | 49 | " | " | intermittent, relapse 270. |
| 38 | 31 | 9 | " | quotidian | intermittent, anticipating. Relapse 25, 1856. |
| 39 | April 1 | 6 | " | " | intermittent at 8 A.M. |
| 40 | 3 | child | | | intermittent. |
| 41 | 4 | 8 | female | tertian | remittent, began as intermittent. |
| 42 | 4 | 2 | " | | intermittent, influenza. |
| 43 | 5 | 38 | male | | intermittent. |
| 44 | 7 | 19 | " | | intermittent, relapse 1, 1856. |
| 45 | 10 | 4 | " | | intermittent, cerebral symptoms. |
| 46 | 12 | 12 | " | quotidian | intermittent, chill 2 P.M. |
| 47 | 12 | 7 | " | " | intermittent, chill 4 P.M. |
| 48 | 16, 21 | 28 | female | | intermittent. |
| 49 | 19 | 24 | male | | " |
| 50 | 24 | 3 mos. | | | " |
| 51 | 25 | 18 " | " | quotidian | intermittent, anticipating. |
| 52 | 29 | 4 yrs. | | " | intermittent, chill 9 A.M. |
| 53 | May 1, 12 | 17 | " | | intermittent, relapse. |
| 54 | 8, 13 | 17 | female | quartan | remittent, costive, mennorrhagia. |
| 55 | 13 | 24 | male | quotidian | intermittent. |
| 56 | 17 | 30 | female | double tertian | intermittent, influenza. |

In cases 14, 15, 17, 18, 19, and 53, of 1856, the sulphate of cinchonæ was administered instead of quinine, and to our perfect satisfaction; indeed, we believe that when the object was to give something to prevent relapse, it seemed more efficient than the quinine itself. Our course though was generally to give quinine to arrest the chills, and then to prescribe the cinchonæ and iron to invigorate the system, so as to prevent a return. During our constant attendance upon the cases of this kind, which occurred during last summer and autumn, it did not appear to us that they were precisely of the character of ordinary intermittent. In a large number of cases the functional impression was much graver than is usually manifested in those complaints in this region of country; in cases of even a plain uncomplicated tertian intermittent, the patient felt sick all the intervening days. There appeared to be disposition, in a large number of cases, (as see tables) for the chills to anticipate their hour of return. While in some cases they became each day longer in their continuance. And, again, in other instances, the chill each day became more indistinct, while the fever was more severe and protracted. In the first class of cases, if neglected, the tendency was to run into what is called congestive chill. While under the second described symptoms, "congestive fever," as we would term it, or fever in which prostration and typhoid symptoms supervened, was apt to be the consequence. In quite a number of cases the whole paroxysm, chill fever and sweat, were jumbled or blended together, as occurred in our neighborhood in 1846 and 1847. In our experience, real rigors or shakes were rare during the past year, indeed, it was a common expression with the patient, "Dr., I don't believe this is the real ague I have got, it's not like anything I ever had before." There were a number of cases in which the chief complaint was of the great oppression of breathing, of "a load like a ton of iron on their stomachs," while a feeble pulse was an almost universal feature of the complaint. Again, the blending together, in some cases, of some of the symptoms of what is called milksickness with the more usual effects accompanying the fever, was an interesting circumstance, as in cases 82, 94, 95, 105, 106, as also in three cases in one house, to which we were called in consultation, in a most malarious spot, (cases which are not noted in these tables.) The attending physician considered them milksickness, and there certainly were present the thirst, burning at the stomach, restlessness, torpor of bowels, and black fœted stools, when stools were obtained, usual in that disease. But still we consider they really belonged to the class of periodic fevers, and were but varieties of congestive fever. (*Query*—is milksickness anything else?) Also, in another case, occurring on the 4th of Sept., and which was the preluding stage of case 76, which was undoubtedly a malignant case of congestive fever, there were the burning in the œsophagus and stomach, thirst, black fœted stools, but there were also *sweat* and diarrhœa.

As to the treatment of the cases recorded in the foregoing table, quinine was the sheet-anchor, but it was in almost all cases combined with morphine and extract of gentian, which we believe adds to the efficacy of the quinine. These were generally administered in the form of a pill, in the proportion, to an adult, of 6 or 7 grains of quinine to 3-10 of a grain of morphine, and about 3 grains of the gentian, given every six hours. We have a great objection to small doses and short intervals. In most cases we believe, especially with quinine, it produces much more excitement, and worries the patient much more, while the effect of the larger dose we think more efficient. In pernicious cases, larger doses were sometimes given, but the interval was generally the same. In such cases especially, we believe opium quite as important as quinine. Calomel, in doses of 5 or 6 grains, or its equivalent of blue mass was prescribed when the alvine discharges indicated a deficient intestinal and hepatic secretion. But we are no believers in the liver pathology, and always eschew the term bilious if we can. Where a purgative was needed we generally preferred castor oil and turpentine after the mercurial. But in a large portion of the cases no calomel or purgatives were given; light nourishing diet being always directed, and the tinct. of mur. of iron when convalescent. In regard to the use of mercury, it appeared to us that there was a peculiar proclivity among our patients to ptyalism, with the use of only small quantities of the medicine, which rendered great caution necessary in its administration. What this peculiarity depended upon, would be difficult to determine. But it occurred to the writer, that, inasmuch as ptyalism is primarily an excited and congested state of the mucous membrane, and that congestion or want of proper capillary action appeared to be the tendency of the disease in question, that the mercury acted upon parts already in a condition of preparation for its own special effects. This may be all idle theorizing, but our attention was so often called to the subject during the sickness of last season, that we were naturally led to speculate on its cause. My colleague, Dr. Harris, informs me he also has noticed this peculiar liability to ptyalism. Dr. Nance, however, has, in his neighborhood, not observed any such tendency. We see it remarked by different writers that the disposition to diarrhœa (which since the first invasion of cholera has appeared to accompany more or less all our disorders) has, during the past season, disappeared. Dr. Haller states, that, in the neighborhood of Vandalia, they have had less diarrhœa and dysentery than usual—Dr. Nance relates, that there was much diarrhœa among children, though but *little* among adults: yet, Dr. McBane, residing at the south of the State, says it has been very prevalent for the past three years, and, from being complicated with hepatic derangement, is usually difficult to eradicate or control—and Dr. Harris, at Ottawa, (in the north,) says that dysentery in the summer months was quite common—and upon examining these tables it will be seen, that, of the cases where any note of the condition of the bowels is made, (and, where none was made, probably nothing peculiar presented,) diarrhœa occurred as a marked symptom in 43 cases; while torpor of the bowels was present in 25 cases. But assuming, as would be probable, that where no note of the presence of diarrhœa is made, that none existed, the testimony would stand only 43 cases of diarrhœa against 313 cases in which it was absent. That, in 13 cases, troublesome hemorrhage took place, sometimes in the form of epistaxis, sometimes from the bowels; while in one case, that of a female after recent delivery, uterine flooding came on with each chill, causing great exhaustion; and in another non-pregnant woman, mennorrhagia accompanied the chills. Of the cases attended, 14 had been under quacks of one denomination or another, generally Steamers or Eclectics, as they call themselves; and these cases were generally among the gravest and most protracted that we met with. As before remarked, in many cases, from the hurry of business, neither sex nor type were recorded; but of those that were noted the following are the proportions of each sex, of each type, for each month of each year:—

| **1855.** | Cases of Quotidian. | Cases of Tertian. | Cases of Quartan. | Males. | Females. |
|---|---|---|---|---|---|
| January, | 2 | 2 | | 2 | 2 |
| February, | | | | | 2 |
| March, | 1 | | | | 1 |
| April, | 3 | 1 | | | 4 |
| | | double | | | |
| May, | 1 | | | 1 | |
| June, | 2 | | | | 2 |
| July, | 10 | 7 | | 7 | 10 |
| August, | 31 | 9 | | 19 | 21 |
| September, | 37 | 18 | | 37 | 28 |
| October, | 63 | 22 | | 45 | 41 |
| November, | 19 | 13 | 2 | 11 | 18 |
| December, | 5 | 4 | | 8 | 8 |
| **1856.** | | | | | |
| January, | 1 | 2 | | 4 | 3 |
| February, | 2 | 4 | 1 | 5 | 7 |
| March, | 5 | 8 | | 10 | 8 |
| April, | 5 | 1 | | 9 | 4 |
| May, | 1 | 1 | 1 | 2 | 2 |
| | | double | | | |
| | 188 | 92 | 4 | 160 | 160 |

From this it will appear, that, of 284 cases in which the type was recorded, 188, were quotidian, and 92 tertian; or within a fraction 2 quotidian to 1 tertian, and only four of the quartan type; among these was one case of double quotidian, two cases of double tertian, and one case of double quartan. That of the cases in which sex is recorded, there were exactly the same number of males as females; that Sept. and Oct. supplied the largest number of cases, Aug. coming next in rank, and Nov. fourth; that of the ten gravest cases, Nos. 24, 25, 32, 38, 54, 58, 69, 76, 135, 186, were of the intermittent type, and two only of those, whereof the types have been recorded, or were developed, were remittent from the first. Cases 54, 55, 56, and 186, simulated cholera in many particulars, and were all in individuals residing in the midst of low alluvial lands, subject to overflow at high water.

One singular feature presents itself in reference to the localities of this endemic. Generally the most malignant as well as the greatest *number* of cases, occur in the low or bottom lands; but, during the past summer, as far as we have been able to ascertain, the river bottoms have suffered least. It appears, indeed, to use the words of Dr. Payne, that "last season the order of things was reversed—there was comparative immunity in the bottoms, but almost universal sickness on the hills." Such was emphatically the case in our own neighborhood. We presume this can be explained only by referring to the previous summer, which had so dessicated the hills that the partial rains and snows of the succeeding winter had never really moistened and cooled them, so that, when the constant showers of last summer fell upon them, instead of being absorbed the heated and hardened sub-soil sent it up in vapor. The effects upon vegetation confirm this view, for contrary to expectation and to the usual effects of a wet summer, the low, wet lands yielded the best crops, the hills in many places being really, notwithstanding the rains, too dry.

In this connection, I insert a

METEOROLOGICAL RECORD, FOR 1855, KEPT BY DR. J. O. HARRIS, OF OTTAWA.

| 1855. | Max. Temperature. | Min. Temperature. | Max. Mean Temp. | Min. Mean Temp. | Monthly Mean Temp. | No. of Rain Storms. | No. of Snow Storms. | DISEASES TREATED. |
|---|---|---|---|---|---|---|---|---|
| Jan. | 62 | 8 | 53.33 | 1.33 | 25.09 | 3 | 4 | pneumonia. |
| Feb. | 44 | 7 | 36 | 2.66 | 18.07 | 4 | 10 | phthisis, croup, pharyngitis. |
| Mar. | 57 | 1 | 45 | 12.66 | 32.68 | 1 | 5 | " " pneumonia. |
| April | 91 | 22 | 78.66 | 31.33 | 55.12 | 8 | | erysipelas, croup, pneumonia, neuralgia. |
| May, | 96 | 41 | 84.66 | 47.67 | 63.16 | 9 | | remittent fever, phthisis. |
| June, | 94 | 44 | 88.33 | 50.66 | 69.45 | 10 | | " intermittents. |
| July, | 95 | 58 | 86.33 | 65 | 74.13 | 9 | | " " |
| Aug. | 93 | 55 | 84 | 58.33 | 70.74 | 10 | | insanity, remittent fever, intermittents, dysentery, cholera infantum. |
| Sept. | 88 | 45 | 78.33 | 53.66 | 67.92 | 12 | | insanity, remittent fever, intermittents, dysentery, cholera infantum. |
| Oct. | 78 | 22 | 66 | 36 | 49.07 | 5 | | pneumonia, phthisis, remittent fever, intermittents, and diarrhœa. |
| Nov. | 48 | 18 | 42.66 | 20 | 34.50 | 2 | 1 | pneumonia, phthisis, remittent fever, intermittents, and diarrhœa. |
| Dec. | 63 | 10 | 53 | 3 | 25 88 | 5 | 3 | intermittent fever, remittent fever, pneumonia, neuralgia. |

There fell 44 inches of snow; did not keep a rain-guage till this year.

The mean temperature at Ottawa for the last three years has been as follows: for 1853, 50.73; 1854, 52.05; 1855, 48.87. The mean for the *last three years united*, 50.55. The observations were made at the hours of 7 A. M., and 2 and 9 P. M.

We trust we have made no errors in copying this interesting table, but we would suggest to gentlemen that when they write for publication, they should be careful to write clearly.

From fevers of a periodic type we naturally turn our attention to that form of fever which, by many, is said to be superseding them, and in regard to which your committee had hoped, as before stated, to have received contributions from different parts, sufficient to throw some light upon the vexed question, of whether typhoid fever is a disease, *sui generis*, or only, as asserted by many, a peculiar condition or grade of periodic fever: the grade or condition being the result of previous causes influencing the constitutional tone of the individual attacked, or in other words, so modifying the vital susceptibility of the body as to cause a different manifestation of *effects*. Much as nitrate of potash, under different external conditions, may act as a refrigerant, a diuretic, or a diaphoretic, or, as indeed, many other agents change the mode of manifesting their effects under different conditions of impressibility of the tissues of the body.

On this side of the question appears one of this committee, Dr. J. O. Harris, who says, speaking of the neighborhood of Ottawa, that all the cases *called* typhoid, that "I have seen have invariably lacked the characteristic features of those diseases. Our fevers (as I view the matter) gradually assume a graver type from September to January, but *always* present unmistakable evidences of intermittent or remittent fever, and usually yield wholly or in part to the use of quinine or its equivalents. When treated according to the views above expressed, they usually recover, but when treated as typhus or typhoid, more than one-half die." But Dr. H. gives the name of the disease and the *opinions formed* of what was seen, but does not supply the detail of the symptoms, of what existed, or the evidence by which alone it appears to us this question of identity can be settled.

It does not appear to your committee that the question of most importance to us at this time, is, whether there is such a disease as typhoid fever, according to the description of systematic authors. But whether *we* have such a disease among ourselves in this State, or whether those who, in a different part of the State, speak or write of a disease by that name, speak of the same assemblage of symptoms.

Now, among our correspondents, Dr. Vincenz says, "To my knowledge there occurred not a single case of typhoid fever." Dr. Haller, of Vandalia, does not so much as name typhoid fever among the sickness that has occurred in his neighborhood. Dr. Payne, of Marshall, describes among the complications of remittent fever, cases where typhoid symptoms supervened, and the treatment required, he says, corresponded very nearly with the directions of Dr. Wood, in his article on "Enteric or Typhoid Fever," while the symptoms certainly were very similar. But these cases began as intermittent or remittent fevers.

Again, Dr. Payne, says "*True typhoid fever* is not a very common disease with us. I have seen several cases during a residence of three years, which have answered well to the descriptions of Drs. Wood and Bartlett;" and as to treatment, he coincides with Dr. Nance, of Lafayette, who remarks, that "if a mild course of treatment is pursued, more reliance is placed on turpentine than any other one remedy. The disease is rather regarded as a self limited disease, and consequently an expectant plan of treatment used." But Dr. Payne does not give the initial symptoms—he does not say how it began. And this, we think, is the question at issue, viz.: whether, as Dr. Davis describes it, the disease begins slowly and insidiously, or whether it begins with chills or shiverings, &c., and afterwards takes on those typhoid symptoms, which, we believe, will be found to be the case with more than one-half the cases described as typhoid.

Dr. Wood, in his Practice, says, "This disease sometimes begins abruptly by a chill, followed by the usual symptoms of fever; but as it occurs in this country, it more frequently comes on insidiously, and increases gradually, so that it is often impossible to fix the precise point of commencement." Now the latter, we suppose, is how it commences in Philadelphia. The mode by chill, as described in the first part of the sentence, the mode in which it commences in some other countries, Europe, for example. The mode as described by Dr. Davis, that of its commencement in Chicago, and from the description of typhoid fever given by Dr. Hathwell, of Cass County, and published in the Report on Practical Medicine to this Society, in 1851. It began in Virginia and Princeton, in Cass County, with "an universal degree of languor, restlessness, attended with lowness of spirits, pain in the back, general weakness, deep sighing, soreness of the muscles, precordial uneasiness, difficult respiration, nausea, *cold clammy sweats*, emitting an unpleasant odor, a constant craving for water, which the stomach rejects." Dr. Davis states, *North-Western Journal*, Nov., 1853, page 429, "when he does take to his bed, his *skin* is *dry*, but only moderately hot." That all the secretions are suspended, "except from the mucous membrane of the ilium." Now, how are we to reconcile all these diversities, not of name only, but of description? Yet Dr. Nance, of Stark County, speaks of typhoid fever as a something definitely and fully established among them for years. We much wish he had extended his copious and valuable letter a little further, and given us in detail the *symptoms and beginnings.* He says, "Typhoid fever occurred here quite malignantly in the year 1848, and during that year and 1849 it seemed to be difficult to manage it. Since that time, the type has been milder and it is managed with much better success." Now we *presume* Dr. Nance *speaks* of a disease such as Dr. Davis, of Chicago, describes as above referred to, thus: "But the typhoid fever, as occurs here, almost invariably steals upon its victim slowly. He first feels dull and indisposed to active mental or physical exertion, his head feels dull, slightly aches, or feels as if bound up tight, he has wandering pains in his back and limbs, occasional liquid discharges from his bowels, his appetite is variable, and his sleep often disturbed. These symptoms gradually increase for one or two weeks before the patient finally gives up and takes to his bed; and when he does so, his *skin is dry*, but moderately hot, his pulse is only 85 to 100, and less forcible than natural, his tongue is covered with a thick dirty white fur, and red along the edges, his intellect is dull and disposed to wander when he is sleeping; all his secretions are diminished, except from the mucous membrane of the ilium, which generally gives rise to from one to six thin evacuations per day; the capillary circulation upon the surface is sluggish, in a word, all the organic actions are like the mind of the patient, dull and indifferent."

Now, in comparing what we *suppose* to be Dr. Nance's account with Dr. Hathwell's, it should be borne in mind Dr. Nance speaks of typhoid fever first becoming endemic among them as in 1848: and Dr. Hathwell, whose description we transcribed above, says it began in Cass County in 1847. Now, the description by Dr. Davis is very graphic, and doubtless accurately true of what he sees; but is it a description of what the term typhoid fever is generally applied to by writers in the West? We suspect not—for ourselves, we must acknowledge that such cases are rare in our experience. We suspect that the symptoms of the following case, extracted from the notes of the Chairman of this Committee, will more fully illustrate the *more legitimate* of the cases usually denominated typhoid fever in the West:—23d January, H. A., aged 18, brunette, been healthy, but has been ailing for several days; has taken Wright's pills, blue pills, castor oil, &c.; has slight chilly feelings, with remitting fever; pulse 100 and quick, but soft and compressible; tongue clean, has abdominal and epigastric tenderness, stools frequent, mere yellow grainy water, feet cold and damp, sweats a little at times. 24th. Pulse quick and variable, eyes somewhat injected, slight headache, tongue as before, less tenderness over abdomen, no stool, anorexia, in night felt a little chilly, in morning had more fever, which continued till 2 P.M., is very sensitive to light and noise. On 26th had remitting fever all day; took oil, and it brought dark, feculent, but liquid stools; pulse 100, small, quick, and soft; tongue as before, headache, face dusky, tenderness in right hypochondrium, urine scanty, has slight sweats at times, face is often flushed, is feeble, dull and indifferent, has abdominal pulsations. These symptoms, with delirium in her sleep, the small watery stools, flushings of the face, almost constant headache, anorexia, and dull indifference, continued till first February, when some slight pneumonic symptoms supervened, but which, during the intensely cold weather, in a not very tight room, might very naturally occur. On 2d February the abdomen was rather tense; but by the 11th she was almost convalescent, when she eat a small piece of ham, and all the symptoms returned, with dry tongue, diarrhœa, and tenderness in right iliac region, which, with amendments and relapses, continued till 8th March, when she recovered. We have omitted to state, that, about one week after taking sick, there were eight small red spots on the lower part of the chest, and that twice during her sickness she had epistaxis, and once dark red blood was noticed in her stools. Now, in this case, quinine did not manifest its usual powers, and we had generally to avoid it, only the mildest medicines could be used; a single blue pill would sometimes purge, in fact to prescribe for symptoms, was generally all that seemed admissable. Yet this was distinctly a remitting fever. Will this be classed as typhoid fever? We expect not—yet we believe it had many more of the characteristics ascribed to that disease than most which, at any rate in the southern part of the State, are described by that name.

But do not these diverse descriptions of the disease under consideration suggest some such ideas as these: that, what is called typhoid fever, is a *condition, and not an entity;* that it manifests itself as the *result* of a certain depressed condition of the vital powers, *sometimes* the results of the slow operation of external morbid causes of no great concentration, at other times more rapidly from a more concentrated cause, and again at other times as the results of disease, fever, for instance, of a remittent or a pneumonic kind; because the long continuance of the disease, or the power of the cause giving rise to that fever, has produced just the same depressed vital condition which unwholesome air or insufficient food do in other cases, and by perhaps a slower and more insidious course. Viewed thus, as a condition, we can easily explain its diversities of manifestation. We but suggest these ideas for reflection; *we* have no satisfactory faith on the subject, and should feel indeed glad to see this discrepancy removed. But we cannot close our remarks on typhoid fever better than by again quoting from Prof. Davis' article in the *North-Western Medical and Surgical Journal*, for Nov. 1853, page 428—that "Few diseases are susceptible of greater variation in their specific or individual characters than fevers, dysenteries, pneumonias, &c.: hence, to make the results of experience valuable to others, it is not sufficient merely to state the medicines given and the proportion of recoveries, *but enough of the actual symptoms must be stated to enable each reader to compare the character of the disease treated with cases of the same class in his own field of practice.*" [The italics are ours.]

[To be concluded in our next issue.]

